# Computational repositioning and preclinical validation of mifepristone for human vestibular schwannoma

**DOI:** 10.1038/s41598-018-23609-7

**Published:** 2018-04-03

**Authors:** Jessica E. Sagers, Adam S. Brown, Sasa Vasilijic, Rebecca M. Lewis, Mehmet I. Sahin, Lukas D. Landegger, Roy H. Perlis, Isaac S. Kohane, D. Bradley Welling, Chirag J. Patel, Konstantina M. Stankovic

**Affiliations:** 1 Eaton-Peabody Laboratories, Department of Otolaryngology, Massachusetts Eye and Ear, Boston, MA 02114 USA; 2 Program in Speech and Hearing Bioscience and Technology, Harvard Medical School, Boston, MA 02115 USA; 3 Department of Biomedical Informatics, Harvard Medical School, Boston, MA 02115 USA; 4 Harvard Program in Therapeutic Science, Harvard Medical School, Boston, MA 02115 USA; 5 Department of Otolaryngology, Harvard Medical School, Boston, MA 02114 USA; 6 Department of Otolaryngology, Vienna General Hospital, Medical University of Vienna, Vienna, 1090 Austria; 7 Center for Experimental Drugs and Diagnostics, Department of Psychiatry and Center for Human Genetic Research, Massachusetts General Hospital, Harvard Medical School, Boston, MA 02114 USA

## Abstract

The computational repositioning of existing drugs represents an appealing avenue for identifying effective compounds to treat diseases with no FDA-approved pharmacotherapies. Here we present the largest meta-analysis to date of differential gene expression in human vestibular schwannoma (VS), a debilitating intracranial tumor, and use these data to inform the first application of algorithm-based drug repositioning for this tumor class. We apply an open-source computational drug repositioning platform to gene expression data from 80 patient tumors and identify eight promising FDA-approved drugs with potential for repurposing in VS. Of these eight, mifepristone, a progesterone and glucocorticoid receptor antagonist, consistently and adversely affects the morphology, metabolic activity, and proliferation of primary human VS cells and HEI-193 human schwannoma cells. Mifepristone treatment reduces VS cell viability more significantly than cells derived from patient meningiomas, while healthy human Schwann cells remain unaffected. Our data recommend a Phase II clinical trial of mifepristone in VS.

## Introduction

Vestibular schwannoma (VS) is the fourth most common intracranial tumor and the most common tumor of the cerebellopontine angle, arising from neoplastic Schwann cells of the vestibular nerve. No drug is FDA-approved to treat VS. In 95% of patients, these tumors cause debilitating sensorineural hearing loss (SNHL) and tinnitus, and can also lead to dizziness and facial paralysis. Bilateral VSs are the hallmark of neurofibromatosis type 2 (NF2), an autosomal dominant disorder caused by inactivation or loss of both alleles of the *NF2* gene. If left untreated, growing VSs can compress the brainstem and lead to death. Mutations in the *NF2* gene are identified in 100% of NF2-associated VSs and 66% of sporadically arising VSs^1^. Though mechanisms of VS-induced SNHL are multifactorial^2,3^, NF2-associated SNHL often correlates with VS size^2,4^. This observation suggests that slowing or inhibiting VS growth may not only prolong a patient’s time to surgical intervention, but also minimize or prevent associated SNHL, substantially improving quality of life. Current treatment options for VS are limited to surgical resection and radiation therapy, both of which carry massive risks for patients, including facial nerve paralysis and loss of hearing. Identification of a drug with the potential to slow or halt VS growth, thereby preserving acoustic hearing and delaying life-threatening complications, represents a major unmet need.

The computational repositioning of FDA-approved drugs, in which data-driven analyses of gene-compound interactions catalyze the pursuit of new indications for approved drugs, provides a transformative avenue for therapeutic innovation^5^. Using novel computational strategies to capitalize on emerging high-throughput genomic information, it is possible to identify safe drugs with evidence-based potential for repurposing in other conditions, which can eliminate the need for a Phase I safety trial and expedite Phase II efficacy trials. Computational drug repositioning represents an appealing approach for diseases with no approved pharmacotherapies, narrowing down a potentially infinite biochemical playing field to a few high-potential candidates.

One approach for surveying drug-based perturbations relies on comparing the RNA-level gene expression profile specific to a given disease to large, pre-generated databases of multi-drug exposure profiles or known gene-drug interactions. The most commonly used tool to interrogate such data is the Broad Connectivity Map (CMAP)^6^, an online platform for matching differentially expressed gene sets from a disease of interest to a library of drug exposure profiles generated from human cell lines. CMAP relies on a modified Kolmogorov-Smirnov enrichment test to rank potentially effective compounds. The natural appeal of its resulting “connectivity score” has led to a large number of CMAP-relevant studies and expansion of these methodologies in targeted disease areas^7–10^. Such studies have led to several high-profile drug repositioning recommendations, such as the well-known case of the anticonvulsant topimarate for use in inflammatory bowel disease (IBD)^11^. Though gene expression data initially indicated that topimarate may be a sensible choice for repositioning in IBD, medical experts pushed back on this result because one of the most frequent severe adverse effects of topimarate is diarrhea, from which IBD patients already disproportionately suffer^12^. Therefore, a holistic evaluation of drug safety within the potential confounds of a new disease indication should never be neglected.

In order to identify an FDA-approved drug with potential for repositioning in VS, we conducted the largest meta-analysis of human VS transcriptomes to date and applied a computational drug repositioning algorithm to match differential gene expression patterns characteristic of VS with known interactions between genes and FDA-approved drugs. Then, after generating a shortlist of drugs with high potential for repositioning, we relied on the clinical expertise of neuro-otologists specializing in VS management to select mifepristone, a progesterone receptor antagonist approved for use in medical abortion, as a candidate worthy of further validation. We use primary human VS cells and immortalized human cell lines to validate mifepristone for repositioning in the treatment of this debilitating tumor. Mifepristone is known to cross the blood-brain barrier and carries a mild side effect profile even in long-term clinical trials. We recommend a Phase II clinical trial of mifepristone in VS.

## Results

### Computational drug repositioning based on differential gene expression reveals drugs with high potential for repurposing in VS

**Figure 1:**
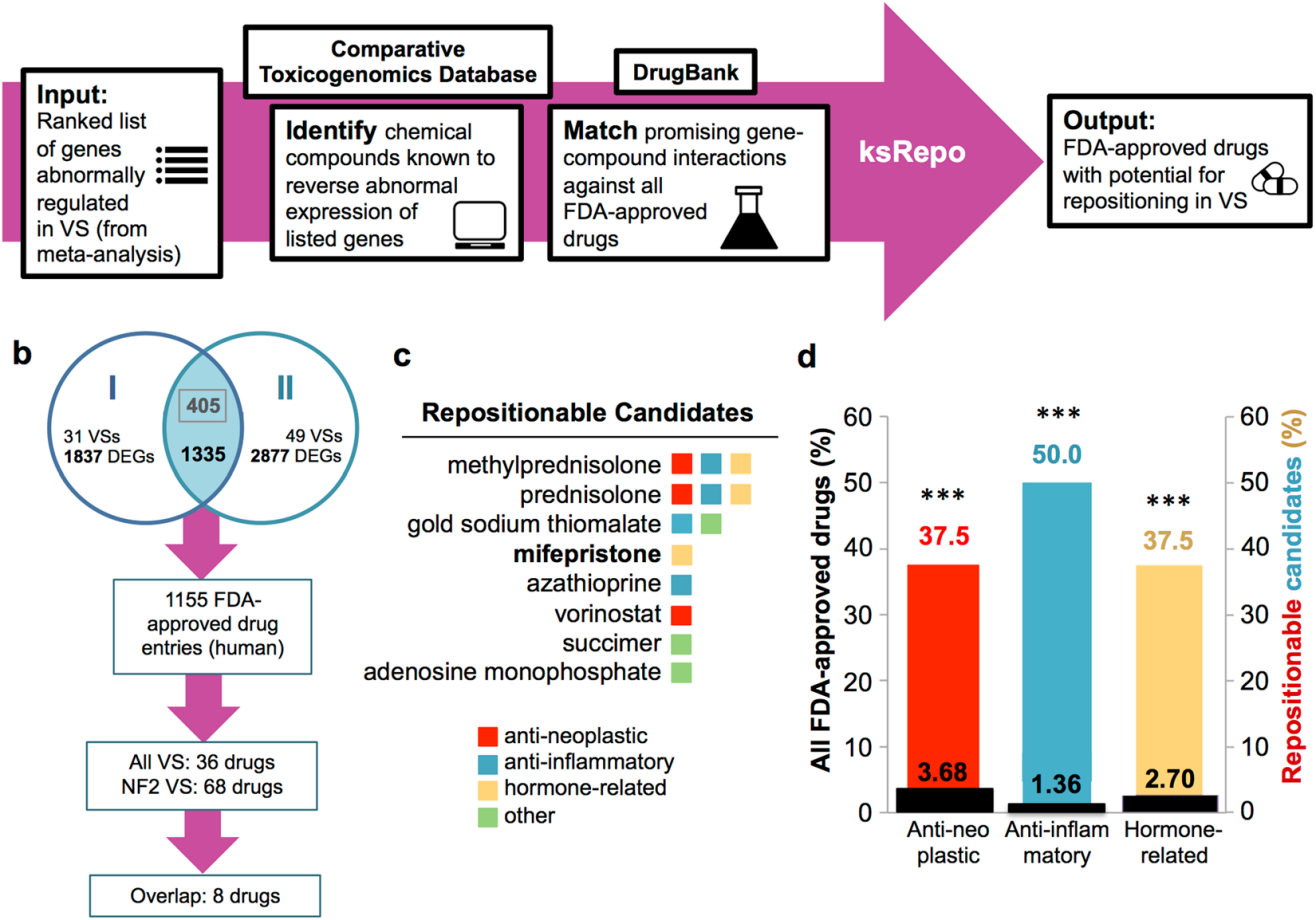
Computational repositioning of FDA-approved drugs using ksRepo. (**a**) ksRepo workflow schematic. (**b**) Largest meta-analysis to date of genome-wide expression in VS (n = 80 tumors), yielding 1,335 commonly dysregulated genes, 405 of which are significantly differentially expressed after Bonferroni correction (p < 0.05); ksRepo recommends 8 drugs with high potential for repositioning in sporadic and NF2-associated VS. (**c**) Drug classes of repositionable candidates from ksRepo analysis. (**d**) Significant enrichment of anti-inflammatory, anti-neoplastic, and hormone-related drugs from 1,155 FDA-approved drugs after ksRepo analysis (***p < 0.001; black bars, percentage of all drugs in DrugBank that fall within each category; colored bars, percentage of drugs on our ksRepo-recommended shortlist that fall within each category).

To identify FDA-approved drugs with potential for repositioning in VS, we conducted a computational screen using the open-source drug repositioning platform ksRepo, developed to screen expression profiles from any microarray or sequencing platform against any available database of gene-drug interactions^13^. ksRepo uses a modified Kolmogorov-Smirnov statistic to compare a ranked list of differentially expressed genes (DEGs) characteristic of a given disease with transcriptional signatures associated with drugs known to interact with those genes, as publicly stored in the Comparative Toxicogenomics Database (CTD)^14^. From that list of drugs, ksRepo selects for compounds with entries in DrugBank, a compendium of FDA-approved drugs^15^. The output is a list of FDA-approved drugs hypothesized to modulate genes with aberrant expression patterns in patients with disease (Fig. 1a). We selected ksRepo for this specific analysis because it does not require input data to be generated via a specific platform (as CMAP does); allows us to choose the database we use (the CTD is manually curated, allowing for high-fidelity associations); affords us flexibility in selecting which drugs to test (rather than relying on pre-compiled, pre-chosen gene signatures); and is compatible with gene-level meta-analysis. ksRepo was recently shown to be successful against a meta-analysis of DEGs from five independent prostate cancer datasets, from which this algorithm successfully predicted significance for five approved therapies in prostate cancer treatment^13^.

To provide robust input to ksRepo, we conducted the largest meta-analysis of primary human VS tissue to date, comprising genome-wide transcriptional microarray data from 80 tumors and 16 control nerves (Fig. 1b). Combined analysis of expression data from two large published datasets, one publicly available (NCBI GEO, GSE39645)^16^ and one published and donated upon request^17^, yields 1,335 genes found to be commonly and concordantly dysregulated in VS, with 405 reaching significance after Bonferroni correction for multiple hypothesis testing (p < 0.05) (Supplementary Data Files S1, S2). ksRepo takes the entire meta-analytic expression profile as input in order to screen a comprehensive picture of tumor-related gene expression against the known interactions of 1,155 FDA-approved drugs. As 16 of 80 VSs in the meta-analysis were harvested from patients with NF2, we also conducted a separate, parallel analysis comprising only NF2-associated tumors. ksRepo returned 36 drugs with potential for repositioning from the complete VS meta-analysis and 68 drugs from the analysis specific to NF2-associated tumors (Supplementary Data Files S3, S4). Though there is no consensus frontline pharmacologic therapy for VS, ksRepo successfully identified multiple classes of drugs that have shown limited efficacy against this tumor, such as non-steroidal anti-inflammatory drugs (NSAIDs), glucocorticoids, tyrosine kinase inhibitors, and histone deacetylase inhibitors (Supplementary Data Files S2, S4).

Eight drugs appeared in both analyses and were prioritized for preclinical validation (Fig. 1c). Out of all FDA-approved drugs, this group of eight demonstrates significant enrichment for anti-inflammatory drugs, hormone-related compounds, and anti-neoplastic agents (Fig. 1d). Specifically, though anti-inflammatory drugs represent just 1.36% of all FDA-approved drugs, four of our eight drugs were classified as anti-inflammatory (50%). Similarly, hormone-related compounds demonstrated a 13.9-fold enrichment (37.5% of recommended drugs from 2.70% of all hormone-related drugs,), and anti-neoplastic agents a 10.2-fold increase (37.5% of recommended drugs from 3.68% of all anti-neoplastic drugs). After thorough theoretical and medical investigation of each of these eight drugs in relation to 1) its medical potential to slow or halt VS growth and 2) its potential to translate safely and effectively to VS patients, mifepristone emerged as the most promising lead candidate.

### Mifepristone reduces metabolic activity and proliferation of HEI-193 human schwannoma cells in culture

Mifepristone (RU486) is a progesterone and glucocorticoid receptor antagonist approved by the FDA for use in medical abortion. This steroid analog is able to cross the blood-brain barrier^18^ and has been shown to provide palliative benefits to patients with other intracranial tumors, such as glioblastoma multiforme^18^ and meningioma^19^. *In vitro*, mifepristone produces antiproliferative effects on cervical^20^, breast^21^, endometrial^22^, ovarian^23^, and prostate cancer cells^24^, regardless of progesterone receptor expression^25^. In human trials, mifepristone administration has been documented to improve quality of life for patients suffering from advanced thymic, renal, colon, leukemic, and pancreatic cancers^26,27^. Long-term administration of oral mifepristone is well tolerated by adults and carries only a mild toxicity profile^28^. Independently of our computational repositioning analysis, when gene expression data from our meta-analysis was input to Ingenuity Pathway Analysis (QIAGEN Inc., https://www.qiagenbioinformatics.com/products/ingenuitypathway-analysis), mifepristone was predicted as a significant upstream regulator of the resulting networks (p = 4.26*10^−5^) and theorized to act upstream of inflammatory markers characteristic of VS, such as TNF and NFkB^29,30^ (Supplementary Fig. S1). We did not perform further experiments to interrogate this hypothesis.

**Figure 2:**
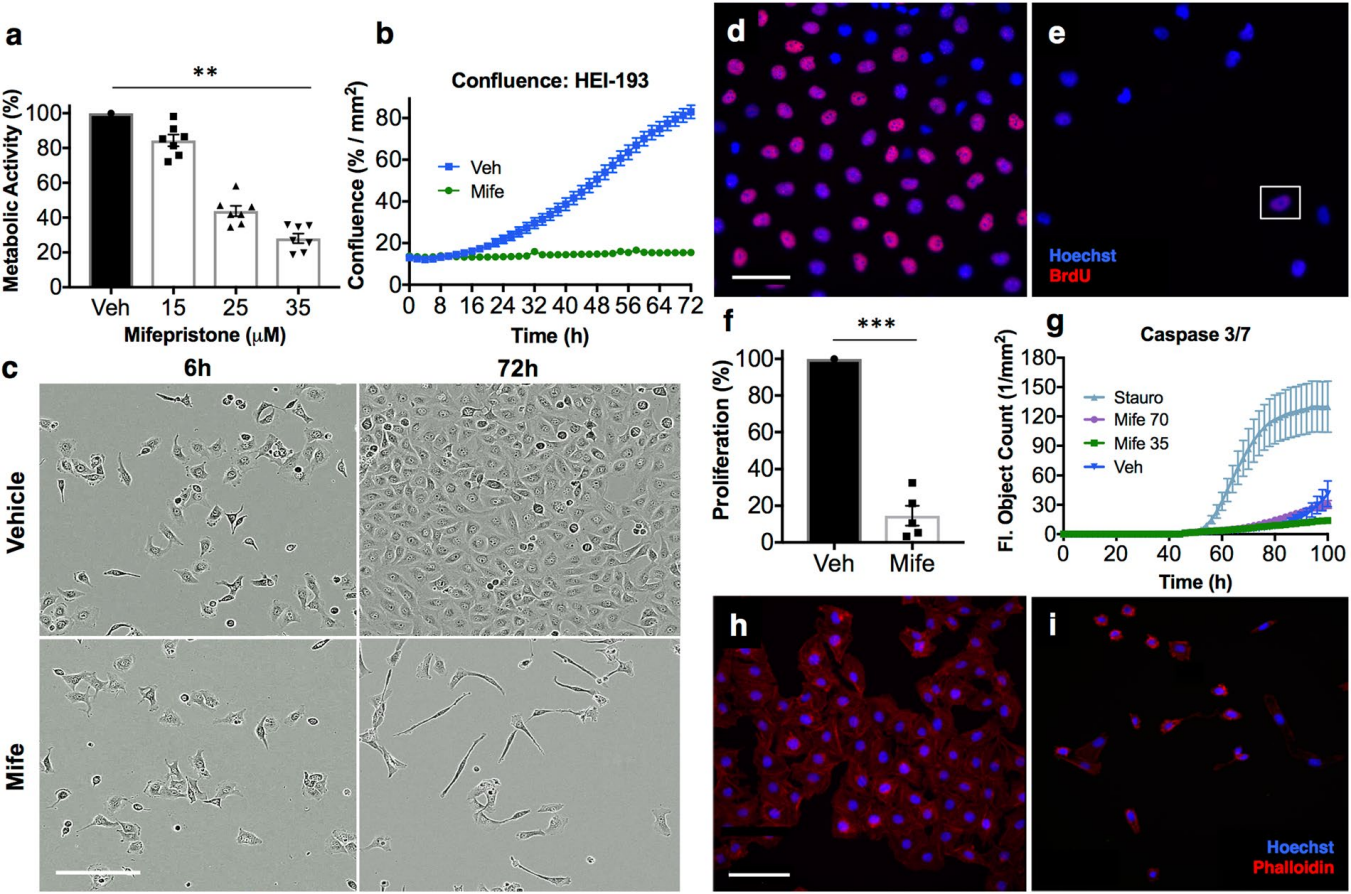
Mifepristone adversely affects HEI-193 cells in culture. (**a**) Metabolic viability of HEI-193 cells decreases with increasing concentration of mifepristone after 72 h in culture. Individual data points represent metabolic activity as percentage of vehicle-treated control for seven independent experiments, performed in replicates of 3–5 wells per condition (vehicle-treated cells versus 35 μM mifepristone-treated cells, p = 0.006; **p < 0.01). (**b**) Phase object confluence remains constant among cells treated for 72 hours with 35 μM mifepristone, while vehicle-treated cells exhibit normal proliferation. Error bars in the mifepristone-treated condition are smaller than the size of the symbol. (**c**) 10X phase contrast images of vehicle-treated cells (top row) and cells treated with 35 μM mifepristone (bottom row) 6 h and 72 h post-treatment. (**d**–**f**) The percentage of BrdU+ HEI-193 cells significantly declines after 72 h treatment with mifepristone (p = 0.0007; ***p < 0.001): (**d**) 25X epifluorescence image of cells treated with 0.1% DMSO vehicle, with BrdU in red and Hoechst stain in blue; (**e**) Cells treated with 35 μM mifepristone, where the white box encloses a single BrdU+ cell; (**f**) Quantification of five replicate experiments. (**g**) Activation of fluorescent caspase 3/7 detected via live-cell imaging over 100 h of mifepristone treatment reveals a slight but not significant elevation in caspase 3/7 activity between mifepristone-treated cells (70 μM and 35 μM) and vehicle-treated control cells; positive control is staurosporine (0.5 μM) and drug is applied 48 h after plating. Note that after 96 h in culture, normally proliferating vehicle-treated cells become overly confluent and begin to apoptose. (**h**–**i**) Phalloidin staining of mifepristone-treated cells reveals crumpled f-actin morphology after 72 h: (**h**) Cells treated with 0.1% DMSO vehicle; (**i**) Cells treated with 35 μM mifepristone, where rhodamine phalloidin is red and Hoechst stain is blue. Center values in histograms are means; error bars are s.e.m.

Administration of mifepristone to HEI-193 immortalized human schwannoma cells in culture produces a significant, dose-dependent response in metabolic activity (Fig. 2a); a significant reduction in cell confluence (Fig. 2b,c); and a dramatic decline in cellular proliferation (Fig. 2d–f) without producing a significant increase in apoptosis (Fig. 2g; Supplementary Fig. S2). Under mifepristone treatment, HEI-193 cells assume a long, thin, spindle-like shape (Fig. 2c; Supplementary Videos S1, S2). This observation is reported in previous studies of mifepristone treatment of ovarian, breast, prostate, and nerve cells, where such morphological changes are attributed to dysregulated distribution of f-actin and tubulin proteins in the cytoskeleton^31^. Continuous, dynamic actin remodeling is characteristic of *NF2*-deficient schwannoma cells^32^, as the *NF2* protein product, merlin, is known to selectively bind f-actin^33^. Phalloidin staining confirms the shrunken, crumpled appearance of f-actin in mifepristone-treated cells (Fig. 2h,i). Enzyme-linked immunosorbent assay for progesterone in conditioned cell culture medium reveals higher levels of progesterone in medium collected from mifepristone-treated cells than from untreated cells, suggesting that mifepristone may compete with progesterone for receptor binding (Supplementary Fig. S3).

### Mifepristone reduces metabolic activity and proliferation of primary human VS cells in culture, regardless of NF2 mutation

**Figure 3:**
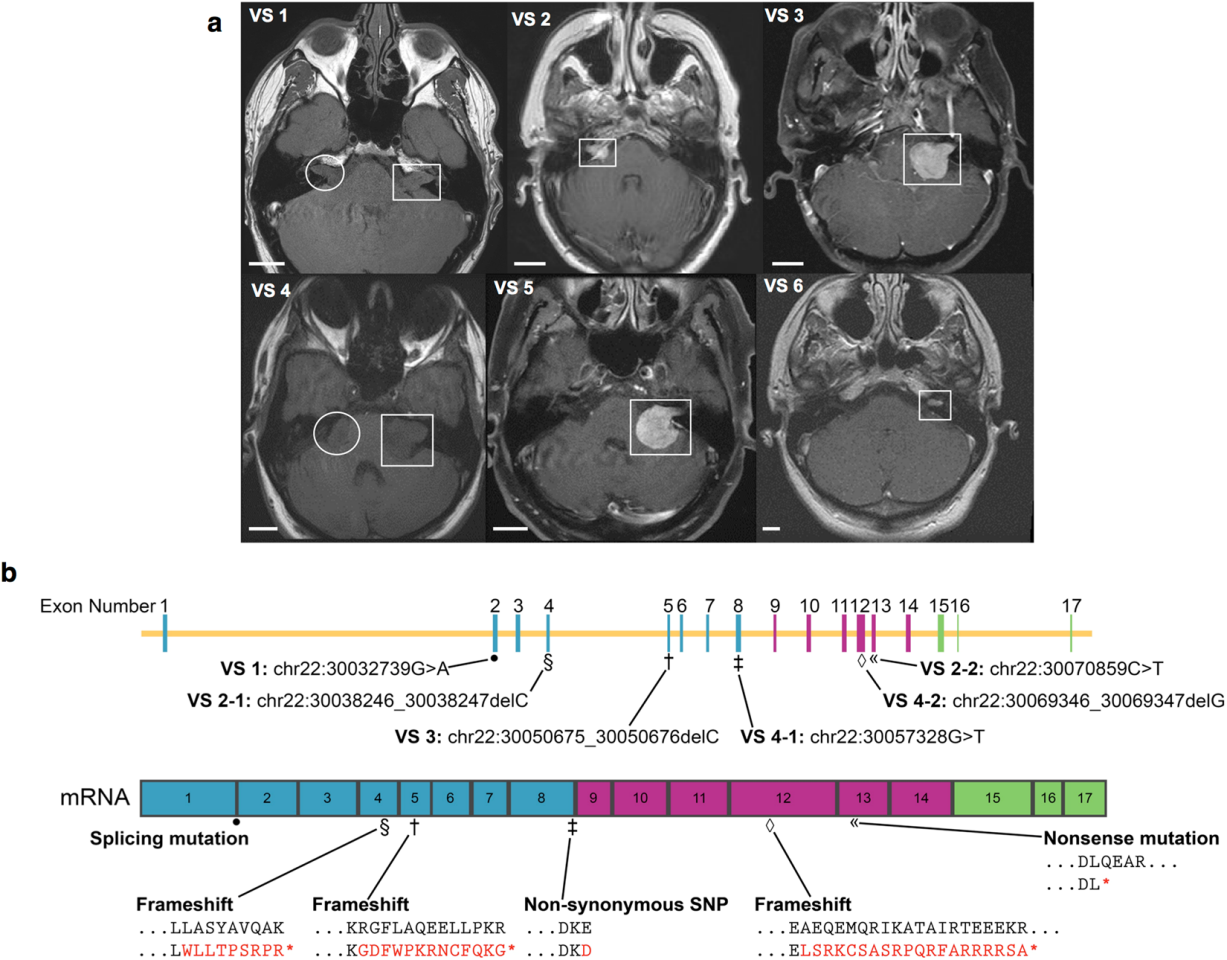
Tumor size and *NF2* mutation type show no relationship with response to mifepristone. (**a**) MRI scans of six VS patients whose primary tumor cells were treated with mifepristone after surgical resection (for scans of four additional, non-sequenced tumors, see Supplementary Fig. S4). White rectangles, VSs; white circles, second (smaller) VSs in NF2 patients, who presented with bilateral VSs. Scale bars, 20 mm. Scans for VS 1 and VS 4 were conducted without the use of contrast agent due to patient intolerance. (**b**) Schematic of *NF2* gene (above) and resulting mRNA (below), describing mutation locus, mutation type, and resulting amino acid change for each tumor in (**a**). Tumors 2 and 4 each contained two mutations in the *NF2* gene, while for tumors 5 and 6, *NF2* mutations were not found.

Current, large-scale meta-analyses of drug toxicology, bioavailability, and efficacy in animal models reveal a shocking lack of predictive power when compared to human data^34,35^. Accordingly, the U.S. National Research Council has recommended the substitution of model animal testing with *in vitro* human cell-based assays and *in silico* modeling of diseases and networks^36^. We evaluated the effect of mifepristone applied directly to primary human VS cells. Fresh VS tissue samples from ten human patients undergoing tumor resection surgery were collected and schwannoma cells grown in the laboratory according to published protocols^37,38^. When we received research tissue in excess of the amount necessary for cell culture, we also performed single-gene sequencing of the *NF2* gene (Fig. 3a). Four of six sequenced VSs exhibited novel mutations in the *NF2* gene, a fraction consistent with published literature^1^ (Fig. 3b; Supplementary Fig. S4).

**Figure 4:**
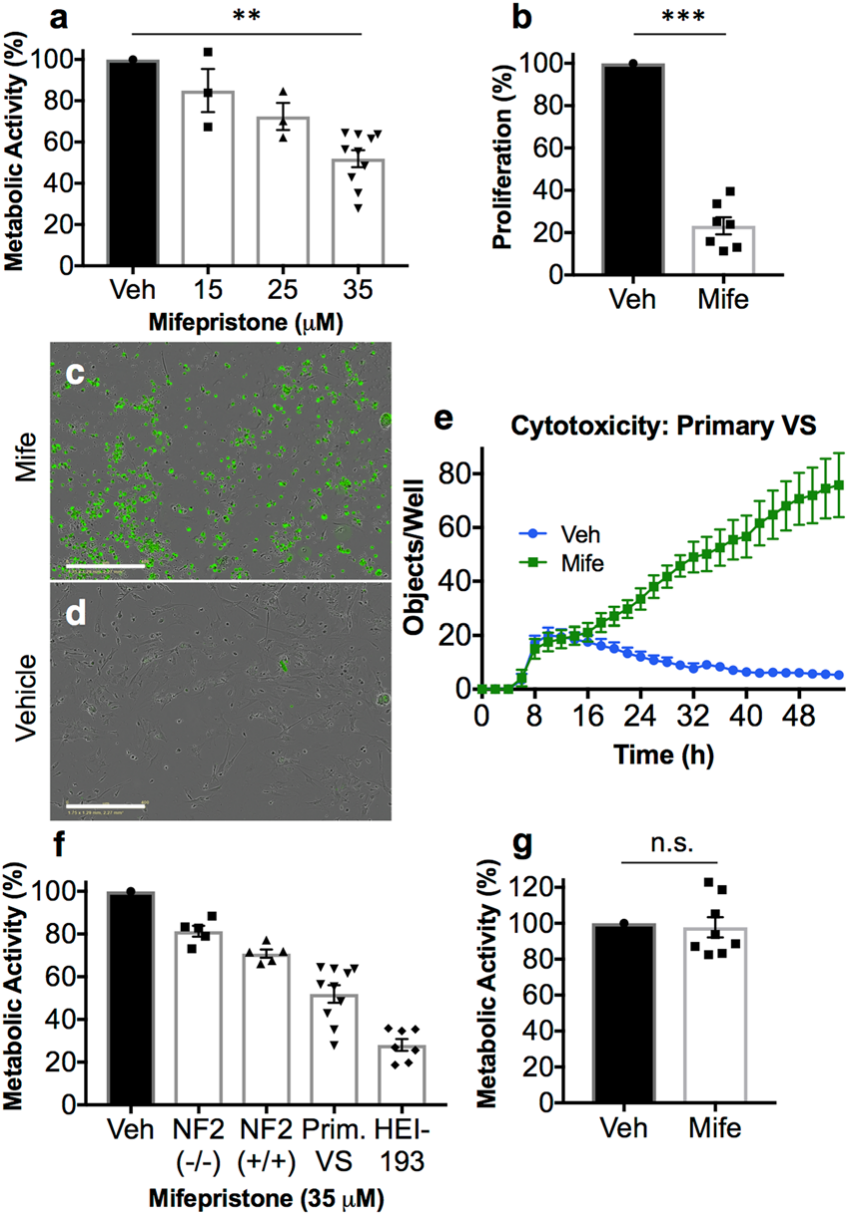
Mifepristone adversely affects primary human VS cells and human-derived arachnoid cells in culture, but leaves primary human Schwann cells unaffected. (**a**) Metabolic activity of primary VS cells declines with increasing concentrations of mifepristone; individual data points represent metabolic activity as percentage of vehicle-treated control for ten individual tumors, performed in replicates of 3–5 wells per condition (vehicle-treated cells versus 35 μM mifepristone-treated cells, p = 0.002; **p < 0.01). (**b**) Quantification of the significant decline in BrdU incorporation observed in primary VS cells treated with 35 μM mifepristone (p = 0.0002; ***p < 0.001). (**c**–**e**) Live cell fluorescence microscopy reveals a significant increase in cytotoxicity of VS cells under mifepristone treatment; representative data from a single tumor, quantified from nine replicate images per treated well, performed in quadruplicate for each treatment condition: (**c**) Primary human VS cells imaged at 10X after treatment with 35 μM mifepristone for 72 hours, where cytotoxicity is indicated via green fluorescent signal; (**d**) Vehicle-treated control cells (DMSO 0.1%); (**e**) Quantification of cytotoxicity, reported as number of green objects per well after thresholding to exclude small cellular debris. (**f**) Mifepristone reduces the metabolic viability of schwannoma cells more significantly than that of AC-CRISPR NF2(−/−) and AC-CRISPR NF2(+/+) human arachnoid cells. (**g**) Mifepristone does not adversely affect the metabolic viability of primary human Schwann cells in culture (n = 8, p = 0.23). Center values in histograms are means; error bars are s.e.m.

When applied to primary human VS cells, mifepristone produced a dose-dependent response in metabolic activity and a dramatic reduction in cellular proliferation (Fig. 4a,b). Live-cell fluorescence imaging revealed a marked increase in cytotoxicity in primary cultures (Fig. 4c–e). No correlation in drug response with tumor size or *NF2* mutation type was observed.

### Mifepristone produces a more significant effect on VS cells than arachnoid cells, but does not adversely affect healthy Schwann cells

In a long-term clinical trial of mifepristone for unresectable meningioma, minor responses resulting in clinical benefit were noted in eight of 28 patients^28^, though a subsequent Phase III trial reported no difference between treatment and placebo^39^. To evaluate the effect of this drug on schwannoma cells in comparison to meningioma cells, we compared mifepristone-treated primary human VS and HEI-193 cells to immortalized human arachnoid cells in which the *NF2* gene has been excised by CRISPR^40^. Primary VS cells responded more dramatically to mifepristone than arachnoid cells with or without the *NF2* gene (Fig. 4f), suggesting that schwannoma cells are more responsive than meningioma cells to this drug.

**Figure 5:**
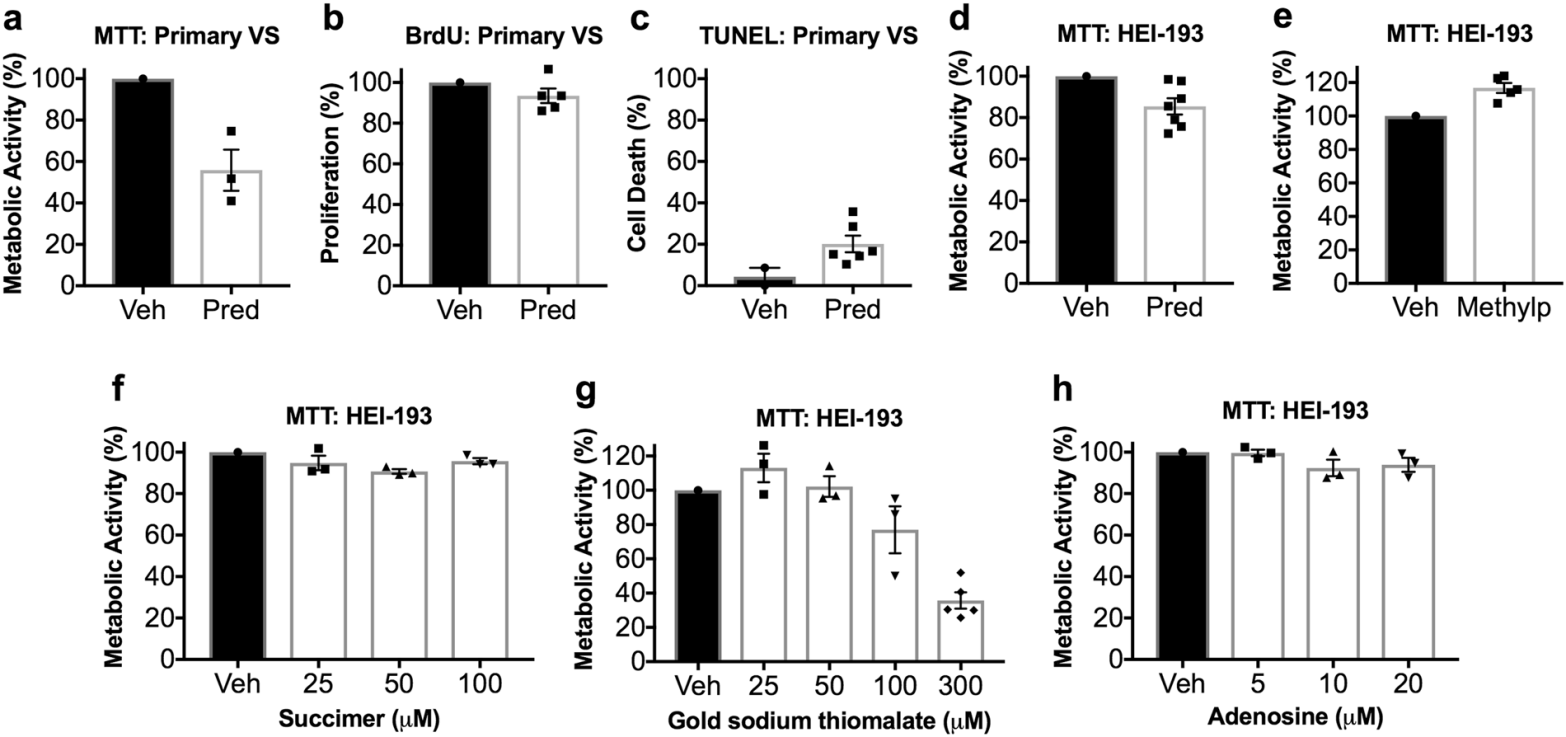
Results of metabolic activity (MTT), proliferation (BrdU incorporation), and cell death (terminal dUTP nick end labeling, TUNEL) assays performed on HEI-193 and primary VS cells treated for 72 h with other drugs recommended by ksRepo. (**a**) Treatment with 25 μM prednisolone produces a modest effect on the metabolic activity of primary VS cells in culture; (**b**) Treatment with 25 μM prednisolone produces no effect on the proliferation of primary VS cells in culture; (**c**) Treatment with 25 μM prednisolone produces a 15.85% elevation in cell death among primary VS cells when compared to vehicle-treated controls; (**d**) Treatment with 25 μM prednisolone produces no significant effect on HEI-193 cell metabolic activity; (**e**) Treatment with 27 μM methylprednisolone produces no significant effect on HEI-193 cell metabolic activity; (**f**) Treatment with 25, 50, and 100 μM succimer produces no significant effect on HEI-193 cell metabolic activity; (**g**) Treatment with 25, 50, 100, and 300 μM gold sodium thiomalate produces a significant effect on HEI-193 cell metabolic activity, but only at clinically unreasonable concentrations; (**h**) Treatment with 5, 10, and 15 μM adenosine monophosphate produces no significant effect on HEI-193 cell metabolic activity.

Additionally, to ensure that mifepristone administration did not lead to adverse effects among healthy human Schwann cells, primary human Schwann cells were cultured from eight great auricular nerves (GANs) harvested from patients undergoing benign parotidectomy or neck dissection^37,38^. Treatment of these cells with mifepristone did not cause appreciable changes in metabolic activity (Fig. 4g). Preliminary testing of clinically reasonable concentrations of other drugs recommended by ksRepo, including adenosine monophosphate, gold sodium thiomalate, succimer, and methylprednisolone, showed no effect on the metabolic activity of HEI-193 cells, though prednisolone produced modest effects when applied to primary human VS cells (Fig. 5a–h).

## Discussion

The *in silico* repositioning of mifepristone for human VS using pooled human transcriptomic data, as well as the preclinical validation of this drug on primary human cells, constitutes a powerful case for the computational identification of novel indications for FDA-approved drugs. Future work will include more detailed pathway analyses of the shared transcriptional responses elicited by all top compounds selected by ksRepo in order to unveil new druggable targets and elucidate additional mechanisms that may drive VS growth. Additionally, as computational drug screening matures, integrating gene expression data with gene mutation data, comprehensive proteomics, pathway analyses, and human exposure data will allow us to refine the quality of algorithm-recommended results, decreasing the odds of false positives and false negatives.

Despite the fact that there is no approved pharmacotherapy for VS, ksRepo successfully identified a variety of drug classes that have shown variable degrees of promise when used against this tumor. Specifically, ksRepo’s recommendation of the NSAID drugs celecoxib, nimeluside, piroxicam, and indomethacin highlights the finding that NSAIDs are cytostatic against VS *in vitro*^30^, and *in vivo*, retrospective studies show that aspirin intake correlates with decreased tumor volume^41,42^. Accordingly, a prospective, randomized clinical trial for aspirin (a drug that did not survive Bonferroni correction for multiple hypothesis testing after ksRepo identification) in VS is set to begin this year. Similarly, the recommendation of prednisolone and methylprednisolone supports the fact that glucocorticoids are the only treatment consistently used with any measure of success against VS-associated sudden SNHL, by mechanisms that remain unknown^43^.

Given the success of tyrosine kinase and histone deacetylase inhibitors in the treatment of other solid neoplasms^44^, it is not surprising that ksRepo spotlights four of these drugs for potential efficacy in VS. Gefitinib is an EGFR/tyrosine kinase inhibitor that showed early promise in the treatment of VS *in vitro*^45^; a retrospective human study of its analog, erlotinib, in NF2 patients did not result in a measurable reduction in tumor volume overall, though three patients reported hearing improvement^46^. Additionally, ksRepo’s recommendation of histone deacetylase inhibitors belinostat, vorinostat, and panobinostat reflects the preclinical success of AR-42, a novel histone deacetylase inhibitor that is the subject of an active clinical trial^47,48^.

Mifepristone belongs to a surprising class of drugs consistently recommended by ksRepo, comprising compounds specific to receptors for the sex hormones estrogen and progesterone, including norgestimate, levonorgestrel, raloxifene, and tamoxifen (Supplementary Data File S2). The existing VS literature contains conflicting reports regarding estrogen and progesterone receptor expression in these tumors, even when measured using nearly identical methods^49–52^. It is unclear what role sex hormone-related signaling might play in VS development or progression. However, a robust body of clinical literature dating back to Harvey Cushing documents the fact that VSs often present during pregnancy or accelerate growth during this period, indicating a potential correlation between sex hormone receptor-mediated signaling and rate of VS growth^53,54^.

Our approach is limited by the fact that ksRepo does not take into account bevacizumab, a monoclonal antibody targeting vascular endothelial growth factor A (VEGF-A). As a biologic drug designed to target a single protein, bevacizumab may not conform well to a drug repositioning approach focusing on genome-wide expression differences, and our 80-tumor meta-analysis reveals that VEGF-A is not significantly upregulated in VSs when compared to GAN controls (Bonferroni-corrected meta-analytic p = 1; Supplementary Data File S1). Regardless, though no human trial of any drug has shown outstanding results against VS, the most promising effects have been associated with bevacizumab. In six retrospective studies conducted among NF2 patients, bevacizumab decreased tumor volume by at least 20% and was associated with a measurable hearing response in most patients^55^. A recent open-label Phase II trial reported radiological responses in six of 14 NF2-associated tumors, with an accompanying hearing response in five^56^. However, the effect of this antibody is often transient, and when generalized across all studies, seems to produce a response in only approximately 50% of patients^55^. Interestingly, bevacizumab does not seem to be clinically effective against NF2-associated meningiomas, in which VEGF pathway upregulation may not be the driving force behind angiogenesis^57^, supporting the hypothesis that this drug may act on tumor vasculature but not VS cells. Uncomfortable administration and serious adverse events observed among long-term users, such as hypertension, renal failure, and hemorrhage, make this drug untenable for multi-year administration, and patients generally resort to surgery^58^.

A drug like mifepristone, which successfully crosses the blood-brain barrier^18^, drastically inhibits human VS cell proliferation, and is documented to have a mild adverse effect profile even when taken daily for up to 13 years^28^ could potentially be administered throughout a patient’s lifetime, prolonging time to surgery or eliminating the need for resection. Few prospective clinical trials are conducted in the primarily surgical management of this debilitating tumor. However, mifepristone is safe, approved for human use, associated with no serious adverse effects known to be compounded by VS, and deserving of further attention in a devastating disease with no FDA-approved therapy. We recommend a Phase II clinical trial of mifepristone in VS.

## Methods

### GEO Dataset Processing

The NCBI GEO dataset used in this study is GSE39645, an Affymetrix Human Gene 1.0 ST chip-based gene expression study of VS which contained data from 28 patients with sporadic VS, 3 patients with NF2-associated VS, and 8 control nerve samples^16^. Data for GSE39645 was accessed through the NCBI GEO portal and analyzed using the integrated GEO2R tool^59^. As input for GEO2R, we classified each sample within a GEO series as either normal tissue or VS tissue. The GEO2R analysis was performed on both the full dataset (sporadic and NF2 combined), and a subset of samples containing only NF2-syndromic schwannomas. GEO2R provides a list of probes and corresponding gene symbols ranked according to their degree of differential expression (as calculated using the *limma* package in R^60^), and includes p-values and t-statistics for differential expression. Following GEO2R analysis, all results were imported into R^61^ and probe-level differential expression was consolidated to gene-level differential expression using a custom pipeline: t-statistic values were converted to Cohen’s d statistic values and standard error values^62^. Resulting values were combined by gene using a fixed effects meta-analysis (as implemented in the *meta.summaries* function from the *rmeta* package in R^63^. Probes without gene annotations were removed from gene-level consolidation. Following consolidation, significantly differentially expressed genes were taken to be those with a Bonferroni-corrected significance of less than 0.05.

### Additional VS Dataset Processing

Raw Affymetrix Human Genome U219 gene expression data (.CEL files) for 36 patients with sporadic VS, 16 patients with NF2 syndrome-associated VS, and 7 control nerves were generously donated by Agnihotri *et al*.^17^. CEL files were loaded into R using the *justRMA* function from the *affy* package in R^64^. *justRMA* is an automated tool that both performs normalization using the Robust Multi-Array Average method^65^ and also automatically annotates all probes in the normalized dataset using the *Org.Hs.eg.db* annotation database package^66^. Normalization was performed on the full dataset and the NF2-associated schwannomas, as above. Mirroring the GEO2R analysis, each normalized dataset was analyzed using *limma* and consolidated to gene-level differential expression using the custom pipeline described above. As above, significantly differentially expressed genes were taken to be those with a Bonferroni-corrected significance of less than 0.05.

### Meta-Analysis of 80 VS Samples and ksRepo Prediction

To robustly determine differential expression between VS and normal tissues, gene-level data from GSE39645^16^ and Agnihotri *et al*.^17^ were meta-analyzed by first removing genes that were not measured in both the Affymetrix Human Gene 1.0 ST chip and the Affymetrix Human Genome U219 chip, and subsequently combining Cohen’s d and standard error values using a fixed-effects meta-analysis (again using *meta.summaries*). Meta-analysis was performed for the full GSE39645 and Agnihotri datasets, as well as for NF2-associated tumors exclusively. Following meta-analysis, the remaining genes were ranked according to their meta-analytic p-values to generate a gene list for further analysis using ksRepo (package available for download at https://github.com/adam-sam-brown/ksRepo, and described in Brown *et al*.^13^. ksRepo is a gene-based drug repositioning method that uses a modified Kolmogorov-Smirnov (KS) statistic to identify promising drug repositioning opportunities. ksRepo requires a database of compound-gene interactions, which are compared with the ranked meta-analytic gene lists from above. For this analysis, the ksRepo built-in Comparative Toxicogenomics Database (CTD) dataset was selected. The CTD provides a curated resource that links small chemical entities to genes (e.g., gene or protein expression influences) from the scientific literature on numerous model organisms and humans^14^. ksRepo contains a subset of the CTD, containing human-derived interactions between 1,268 unique drugs and 18,041 unique human genes. Drugs in the CTD subset were chosen based on case-insensitive matches between CTD names and names/synonyms for FDA-approved drugs downloaded from DrugBank^15^. The ksRepo output provides both the resampled p-value and FDR value. For the full dataset ksRepo analysis and the NF2-only ksRepo analysis, significant compounds were those for which the FDR was less than 0.05.

### Human Specimen Collection and Primary Cell Culture

Surgical VS and GAN specimens were collected and processed according to protocols approved by the Human Studies Committee of Massachusetts General Hospital and Massachusetts Eye and Ear (Board Reference #14-148 H). Written informed consent was obtained from all subjects prior to inclusion in this study and all procedures were conducted in accordance with the Helsinki Declaration of 1975. Detailed methods for human surgical specimen collection, processing, and culture are previously published^37,38^. VS specimens were harvested from patients undergoing surgical tumor resection, and GAN specimens from healthy patients undergoing benign parotidectomy or neck dissection surgery, during which the GAN is routinely sacrificed. Patients who had received radiation therapy prior to surgery were excluded. After surgical resection, VS or GAN tissue was immediately placed in saline solution, transported to the laboratory, and cultured according to published protocols (Supplementary Methods). All downstream procedures were performed on primary cell cultures or collected culture medium up to but not exceeding two weeks of age in culture to ensure maximal Schwann or schwannoma cell purity^37^.

### HEI-193 and Arachnoid Cell Culture

HEI-193 cells are derived from an NF2 patient with spontaneous bilateral vestibular schwannomas and a history of meningioma; these cells express a splice variant of the merlin protein (encoded by the *NF2* gene), but neither typical isoform^67^. HEI-193 cells were cultured in DMEM/F12-containing medium with 10% fetal bovine serum and 1% penicillin and streptomycin mix. Immortalized NF2-null and NF2-expressing arachnoid AC-CRISPR cell lines derived from primary human autopsy specimens were obtained via generous gift from Dr. Vijaya Ramesh at Massachusetts General Hospital^40^. NF2-null and NF2-expressing arachnoid cells were cultured in DMEM with 15% fetal bovine serum and 1% penicillin and streptomycin mix. All cell lines were maintained in an incubator at 37 degrees Celsius with 5% carbon dioxide and treated with drugs 24-36 hours after seeding at between 15,000-25,000 cells per well in 24-well plates. Phase contrast photos of healthy and drug-treated cultures were taken at 10X magnification on an IncuCyte S3 instrument (Essen Bio).

### Drug Preparation and Treatment

Primary VS and GAN cultures were treated with mifepristone (Sigma Aldrich, lot #WXBC0031V). Fifteen, 25, 35, and 70 μM mifepristone were prepared by suspending the appropriate amount of drug (in powder form) in dimethyl sulfoxide (DMSO). The resulting drug suspension was diluted in culture medium to the concentration of interest, and drug-containing medium was applied to primary VS, GAN, and HEI-193 cells such that the amount of DMSO applied to cells in culture did not exceed 0.1% (24-well plate, 1 mL medium per well). Cultures were incubated with drug-containing medium or 0.1% DMSO vehicle for 72 hours and then processed for downstream applications.

### Proliferation Assay

5′bromo-2′-deoxyuridine (BrdU) was added to label proliferating cells in culture 2 hours before fixation in 4% formalin (paraformaldehyde). Cell membranes were permeabilized with 10 minutes of incubation in 1% Triton X-100 and nuclear membranes with 20 minutes in 2N hydrochloric acid (HCl). Cells were blocked in 5% normal horse serum (NHS) and 1% Triton X-100 and incubated with a primary antibody against BrdU (#OBT0030G, AbD Serotec) overnight, followed by incubation with fluorescent anti-rat immunoglobulin G (AlexaFluor, Life Technologies). Cells were stained with Hoechst 33342 (Invitrogen) and phalloidin/f-actin (ThermoFisher Scientific) and coverslips mounted on slides with VectaShield (Vector Laboratories). The ratio of BrdU-positive to Hoechst-positive nuclei was determined by sampling three random fields of view using a Leica epifluorescence microscope. Manual counts were performed by J.E.S., who was blinded to treatment conditions.

### Metabolic Activity Assay

The metabolic activity of primary VS and HEI-193 cells was assessed using the colorimetric 3-(4,5-dimethylthiazol-2-yl)-2,5-diphenyltetrazolium bromide (MTT) assay (Life Technologies) according to manufacturer’s instructions. Optical density (OD) of each well at 570 nm was read using a spectrophotometer. The average OD value of cells exposed to vehicle (0.1% DMSO) was set to 100% and used to normalize OD values of cells treated with drugs; metabolic activity was then reported as percent change. All statistical testing was performed on raw OD values (see *Statistical Analysis*).

### Flow Cytometry

Apoptotic cell death was assessed using an Annexin V/propidium iodide (PI) staining kit (Miltenyi Biotec). Briefly, HEI-193 cells were seeded into T25 flasks and treated with mifepristone or DMSO vehicle in culture medium for 24 hours as described above. Adherent cells were collected by trypsinization, and non-adherent (floating) cells were collected from culture medium. Cells were centrifuged, washed in PBS, and incubated in 1X annexin binding buffer, annexin V-fluorescein isothiocyanate (FITC), and propidium iodide (PI) according to the manufacturer’s recommendations. Stained cells were immediately analyzed using a Cytomics FC500 flow cytometer. Data were analyzed using CXP Analysis software (version 2.2, Coulter).

### Cell Cycle Analysis

Harvested HEI-193 cells were washed in PBS and fixed in cold 70% ethanol at −20 °C for 18–72 h. Before staining with propidium iodide (PI), cells were centrifuged and washed again in cold PBS. 2 × 10^6^ or fewer cells were incubated with 500 µl staining solution (0.1% Triton X-100 (Sigma), 2 mg/ml RNase A (Qiagen), and 1 µg/ml PI (Miltenyi Biotec) in PBS) for 15 minutes at 37 °C. In order to exclude DNAse activity, RNase A was boiled for 5 minutes and cooled down before its addition to staining solution. Cells were analyzed on a Cytomics FC500 flow cytometer using CXP Analysis software (version 2.2, Coulter).

### Enzyme-linked Immunosorbent Assay

Cell-conditioned medium was collected from mifepristone-treated and untreated HEI-193 cells after 72-hour incubation with the drug. Enzyme-linked immunosorbent assay (ELISA) was performed on each sample in triplicate to assess the quantity of progesterone in cell-conditioned medium, according to the manufacturer’s protocol (Enzo Life Science, #ADI-900-011).

### Cytotoxicity and Cell Confluence Assays

Cytotoxicity and cell confluence were measured using live-cell, time-lapse phase contrast and fluorescence imaging acquired at 10X by an IncuCyte S3 instrument (Essen Bioscience). Cytotoxicity was measured by incorporation of IncuCyte Cytotox Reagent (Essen Bioscience), applied according to manufacturer’s instructions; the reagent fluoresces when it binds DNA after compromise of membrane integrity. Nine images were acquired per well every 2 hours for 72 hours, and cytotoxicity at each time point is reported as number of fluorescent objects per well, after thresholding to avoid the inclusion of small cellular debris. Phase object confluence was measured using time-lapse phase contrast imaging acquired at 10X by the same instrument, analyzing 9 images per well every 2 hours for 72 hours, and is reported as percent confluence per square millimeter of well space.

### Ingenuity Pathway Analysis

Ingenuity Pathway Analysis software (Qiagen) was used to perform standard Core Analysis on all genes in our meta-analysis that reached significance (p < 0.05) after Bonferroni correction for multiple hypothesis testing. Relevant upstream regulators for the resulting networks were identified and analyzed using publicly available Ingenuity Pathway Analysis documentation (Qiagen).

### gDNA Extraction

Genomic DNA (gDNA) was extracted from six vestibular schwannoma tissue samples using the DNeasy Blood and Tissue Kit (Qiagen) following manufacturer’s specifications. The concentration of double-stranded DNA in each sample was evaluated using a Qubit dsDNA BR Assay Kit. A minimum measurement of 50 ng/μl was required for each sample to be included with HaloPlex target enrichment.

### Library Preparation and Targeted Capture

A library of DNA restriction fragments from all coding exons, introns, and UTRs (5′ and 3′) of the NF2 gene was prepared using a HaloPlex HS target enrichment kit (Agilent Technologies), following the manufacturer’s instructions. The total region size was 95.045 kbp with an actual analyzed target of 89.408 kbp bases, which required 2581 amplicons to achieve this 94.07% coverage with maximum validation stringency. Enrichment was performed according to the supplier’s protocol by the Ocular Genomics Institute at Massachusetts Eye and Ear (Boston, MA, USA) (Supplementary Methods).

### NF2 Gene Sequencing

Targeted enrichment sample sequencing was performed on an Illumina MiSeq NGS platform (Illumina, Inc.) by the Next Generation Sequencing Core of the Massachusetts Eye and Ear Ocular Genomics Institute. The purified and individually tagged amplicon libraries for each sample were pooled equimolarly, and a percentage of an internal control (ECD) was added to validate the DNA sequencing and to help balance the overall lack of sequence diversity. The sample pool was then placed in a MiSeq Reagent kit version 2 500-cycle cartridge (Illumina) containing sequencing reagents, and sequencing was performed on the Illumina MiSeq instrument by using a MiSeq Reagent Kit v2 flow cell (Illumina). Raw data were processed and variants prioritized according to best practices of the core facility (Supplementary Methods).

### Statistical analysis

Throughout this paper, though figures present metabolic activity and cellular proliferation data as percentage of vehicle-treated control, all statistical analyses were performed on raw data, in accordance with good statistical practice in pharmacology^68^. Specifically, in Figs 2a and f, 4a,b and g, raw vehicle-treated and mifepristone-treated cell data are compared using two-way (“randomized block”) ANOVA, selected to minimize within-experiment variation by “blocking” treatment data with control data while meeting the equal-variance assumptions required by ANOVA^68^. In Fig. 2a, comparison between control group and mifepristone-treated group (35 μM) was conducted using randomized block ANOVA on mean optical density values per treatment condition measured in eight independent experiments (p = 0.006, F = 42.46, DF = 1). For Fig. 2f, randomized block ANOVA was performed between the ratio of BrdU+ to Hoescht+ cells per treatment condition in five independent experiments (p = 0.0007, F = 88.25, DF = 1). For Fig. 4a, comparison between control group and mifepristone-treated group (35 μM) was conducted using randomized block ANOVA on mean optical density values per treatment condition measured in ten independent experiments (p = 0.002, F = 23.02, DF = 1). In Fig. 4b, randomized block ANOVA was performed between the ratio of BrdU+ to Hoescht+ cells per treatment condition in seven independent experiments (p = 0.0002, F = 68.47, DF = 1). In Fig. 4g, randomized block ANOVA was performed on mean optical density values per treatment condition measured in eight independent experiments (p = 0.230, F = 1.255, DF = 1).

## Electronic supplementary material

Supplementary Information

Supplementary Video S1

Supplementary Video S2

Supplementary Data File S1

Supplementary Data File S2

Supplementary Data File S3

Supplementary Data File S4

## Data Availability

The results of the 80-sample combined VS meta-analysis, 16-sample NF2-specific VS meta-analysis, and ksRepo results derived therefrom are available from FigShare at 10.6084/m9.figshare.4902884. Raw data from the *NF2* gene sequencing analysis are available from FigShare at 10.6084/m9.figshare.4978136.

## References

[1] Hadfield KD (2010). Rates of loss of heterozygosity and mitotic recombination in NF2 schwannomas, sporadic vestibular schwannomas and schwannomatosis schwannomas. Oncogene.

[2] Asthagiri AR (2012). Mechanisms of hearing loss in neurofibromatosis type 2. PLoS ONE.

[3] Dilwali S, Landegger LD, Soares VYR, Deschler DG, Stankovic KM (2015). Secreted factors from human vestibular schwannomas can cause cochlear damage. Sci. Rep..

[4] Plotkin SR, Merker VL, Muzikansky A, Barker FG, Slattery W (2014). Natural history of vestibular schwannoma growth and hearing decline in newly diagnosed neurofibromatosis type 2 patients. Otol. Neurotol..

[5] Li J (2016). A survey of current trends in computational drug repositioning. Brief. Bioinformatics.

[6] Lamb J (2006). The Connectivity Map: using gene-expression signatures to connect small molecules, genes, and disease. Science.

[7] Sirota M (2011). Discovery and preclinical validation of drug indications using compendia of public gene expression data. Sci. Transl. Med..

[8] Pacini C (2013). DvD: An R/Cytoscape pipeline for drug repurposing using public repositories of gene expression data. Bioinformatics.

[9] Duan Q (2014). LINCS Canvas Browser: interactive web app to query, browse and interrogate LINCS L1000 gene expression signatures. Nucleic Acids Res..

[10] Musa, A., *et al*. A review of connectivity map and computational approaches in pharmacogenomics. *Brief. Bioinform*. bbw12 (2017). 10.1093/bib/bbx023 PMC6113891 28334173

[11] Dudley JT (2011). Computational repositioning of the anticonvulsant topimarate for inflammatory bowel disease. Sci. Transl. Med..

[12] Deftereos, S. N. Is a single type of data sufficient for computational drug repositioning? eLetters to *Sci*. *Transl*. *Me*d. (2011): commentary on Dudley, J.T. *et al* (2011).

[13] Brown AS, Kong SW, Kohane IS, Patel CJ (2016). ksRepo: a generalized platform for computational drug repositioning. BMC Bioinformatics.

[14] Davis AP (2015). The comparative toxicogenomics database’s 10th year anniversary: update 2015. Nucleic Acids Res..

[15] Wishart DS (2006). DrugBank: a comprehensive resource for *in silico* drug discovery and exploration. Nucleic Acids Res..

[16] Torres-Martin M (2013). Microarray analysis of gene expression in vestibular schwannomas reveals SPP1/MET signaling pathway and androgen receptor deregulation. Int. J. Oncol..

[17] Agnihotri S (2014). Gene-expression profiling elucidates molecular signaling networks that can be therapeutically targeted in vestibular schwannoma. J. Neurosurg..

[18] Check JH, Wilson C, Cohen R, Sarumi M (2014). Evidence that mifepristone, a progesterone receptor antagonist, can cross the blood brain barrier and provide palliative benefits for glioblastoma multiforme grade IV. Anticancer Res..

[19] Touat M, Lombardi G, Farina P, Kalamarides M, Sanson M (2014). Successful treatment of multiple intracranial meningiomas with the antiprogesterone receptor agent mifepristone (RU486). Acta Neurochir (Wien).

[20] Segovia-Mendoza M (2015). Antihormonal agents as a strategy to improve the effect of chemo-radiation in cervical cancer: *in vitro* and *in vivo* study. BMC Cancer.

[21] Gaddy VT (2004). Mifepristone induces growth arrest, caspase activation, and apoptosis of estrogen receptor-expressing, antiestrogen-resistant breast cancer cells. Clin. Cancer Res..

[22] Moe BT, Vereide AB, Orbo A, Jaeger R, Sager G (2009). Levonorgestrel, medroxyprogesterone and progesterone cause a concentration-dependent reduction in endometrial cancer (Ishikawa) cell density, and high concentrations of progesterone and mifepristone act in synergy. Anticancer Res..

[23] Rose FV, Barnea ER (1996). Response of human ovarian carcinoma cell lines to antiprogestin mifepristone. Oncogene.

[24] Lin MF, Kawachi MH, Stallcup MR, Grunberg SM, Lin FF (1995). Growth inhibition of androgen-insensitive human prostate carcinoma cells by a 19-norsteroid derivative agent, mifepristone. Prostate.

[25] Tieszen CR, Goyeneche AA, Brandhagen BN, Ortbahn CT, Telleria CM (2011). Antiprogestin mifepristone inhibits the growth of cancer cells of reproductive and non-reproductive origin regardless of progesterone receptor expression. BMC Cancer.

[26] Check JH, Dix E, Cohen R, Check D, Wilson C (2010). Efficacy of the progesterone receptor antagonist mifepristone for palliative therapy of patients with a variety of advanced cancer types. Anticancer Res..

[27] Check JH, Check D, Cohen R, Sarumi M (2014). Mifepristone causing complete remission of rapidly advancing leukemia with measurement of progesterone-induced blocking factor. Anticancer Res..

[28] Grunberg SM (2006). Long-term administration of mifepristone (RU486): clinical tolerance during extended treatment of meningioma. Cancer Invest..

[29] Dilwali S (2015). Preclinical validation of anti-nuclear factor kappa B therapy to inhibit human vestibular schwannoma growth. Molecular Oncology.

[30] Dilwali S, Kao S, Fujita T, Landegger LD, Stankovic KM (2015). Nonsteroidal anti-inflammatory medications are cytostatic against human vestibular schwannomas. Transl. Res..

[31] Brandhagen BN (2013). Cytostasis and morphological changes induced by mifepristone in human metastatic cancer cells involve cytoskeletal filamentous actin reorganization and impairment of cell adhesion dynamics. BMC Cancer.

[32] Flaiz C, Kaempchen K, Matthies C, Hanemann CO (2007). Actin-rich protrusions and nonlocalized GTPase activation in merlin-deficient schwannomas. J. Neuropathol. Exp. Neurol..

[33] James MF, Manchanda N, Gonzalez-Agosti C, Hartwig JH, Ramesh V (2001). The neurofibromatosis 2 protein product merlin selectively binds F-actin but not G-actin, and stabilizes the filaments through a lateral association. Biochem. J..

[34] Denayer T, Stöhr T, Van Roy M (2014). Animal models in translational medicine: Validation and prediction. New Horizons in Translational Medicine.

[35] Shanks N, Greek R, Greek J (2009). Are animal models predictive for humans?. Philos. Ethics Humanit. Med..

[36] Krewski D (2010). Toxicity testing in the 21st century: a vision and a strategy. J Toxicol. Environ. Health B. Crit. Rev..

[37] Dilwali S (2014). Primary culture of human Schwann and schwannoma cells: improved and simplified protocol. Hear. Res..

[38] Landegger LD (2017). A unified methodological framework for vestibular schwannoma research. J Vis Exp.

[39] Ji Y (2015). Double-blind phase III randomized trial of the antiprogestin agent mifepristone in the treatment of unresectable meningioma: SWOG S9005. J. Clin. Oncol..

[40] Beauchamp RL (2015). A high-throughput kinome screen reveals serum/glucocorticoid-regulated kinase 1 as a therapeutic target for NF2-deficient meningiomas. Oncotarget.

[41] Kandathil CK (2014). Aspirin intake correlates with halted growth of sporadic vestibular schwannoma *in vivo*. Otol. Neurotol..

[42] Kandathil CK, Cunnane ME, McKenna MJ, Curtin HD, Stankovic KM (2016). Correlation between aspirin intake and reduced growth of human vestibular schwannoma: volumetric analysis. Otol. Neurotol..

[43] Aronzon A, Ruckenstein MJ, Bigelow DC (2003). The efficacy of corticosteroids in restoring hearing in patients undergoing conservative management of acoustic neuromas. Otol. Neurotol..

[44] Arora A, Scholar EM (2005). Role of tyrosine kinase inhibitors in cancer therapy. J. Pharmacol. Exp. Ther..

[45] Curto M, Cole BK, Lallemand D, Liu C, McClatchey AI (2007). Contact-dependent inhibition of EGFR signaling by Nf2/Merlin. J. Cell Biol..

[46] Plotkin SR (2010). Erlotinib for progressive vestibular schwannoma in neurofibromatosis 2 patients. Otol. Neurotol..

[47] Jacob A (2012). Preclinical validation of AR42, a novel histone deacetylase inhibitor, as treatment for vestibular schwannomas. Laryngoscope.

[48] Welling, D. B. Exploratory evaluation of AR-42 histone deacetylase inhibitor in the treatment of vestibular schwannoma and meningioma. ClinicalTrials.gov; identifier #NCT02282917 (2017).

[49] Patel AK, Alexander TH, Andalibi A, Ryan AF, Doherty JK (2008). Vestibular schwannoma quantitative polymerase chain reaction expression of estrogen and progesterone receptors. Laryngoscope.

[50] Cafer S (2008). Expression and clinical significance of Ki-67, oestrogen and progesterone receptors in acoustic neuroma. J. Laryngol. Otol..

[51] Dalgorf DM, Rowsell C, Bilbao JM, Chen JM (2008). Immunohistochemical investigation of hormone receptors and vascular endothelial growth factor concentration in vestibular schwannoma. Skull Base.

[52] Jaiswal S, Agrawal V, Jaiswal AK, Pandey R, Mahapatra AK (2009). Expression of estrogen and progesterone receptors in vestibular schwannomas and their clinical significance. J. Negat. Results Biomed..

[53] Cushing, H. *Tumors of the nervus acusticus and the syndrome of the cerebellopontile angle* (WB Saunders, 1917).

[54] Shah KJ, Chamoun RB (2014). Large vestibular schwannomas presenting during pregnancy: management strategies. J. Neurol. Surg. B. Skull Base.

[55] Goutagny, S. & Kalamarides, M. Medical treatment in neurofibromatosis type 2. Review of the literature and presentation of clinical reports. *Neurochirurgie* (2017). 10.1016/j.neuchi.2016.09.004 28162254

[56] Blakeley JO (2016). Efficacy and biomarker study of bevacizumab for hearing loss resulting from neurofibromatosis type 2-associated vestibular schwannomas. J. Clin. Oncol..

[57] Nunes FP (2013). Bevacizumab treatment for meningiomas in NF2: a retrospective analysis of 15 patients. PLoS ONE.

[58] Slusarz KM, Merker VL, Muzikansky A, Francis SA, Plotkin SR (2014). Long-term toxicity of bevacizumab therapy in neurofibromatosis 2 patients. Cancer Chemother. Pharmacol..

[59] Barrett T (2013). NCBI GEO: archive for functional genomics data sets–update. Nucleic Acids Res..

[60] Ritchie ME (2015). limma powers differential expression analyses for RNA-sequencing and microarray studies. Nucleic Acids Res..

[61] Development Core Team R. R: A language and environment for statistical computing. Vienna, Austria: the R Foundation for Statistical Computing (2011).

[62] Friedman, H. Simplified determinations of statistical power, magnitude of effect and research sample sizes. *Educ Psychol Meas* (1982).

[63] Lumley, T. rmeta: Meta-analysis. R package (2009).

[64] Gautier L, Cope L, Bolstad BM, Irizarry R (2004). A. affy–analysis of Affymetrix GeneChip data at the probe level. Bioinformatics.

[65] Irizarry RA (2003). Exploration, normalization, and summaries of high density oligonucleotide array probe level data. Biostatistics.

[66] Carlson, M. org.Hs.eg.db: Genome wide annotation for human https://bioconductor.org/packages/release/data/annotation/html/org.hs.eg.db.html (2016).

[67] Lepont P (2008). Point mutation in the NF2 gene of HEI-193 human schwannoma cells results in the expression of a merlin isoform with attenuated growth suppressive activity. Mutat. Res..

[68] Lew M (2007). Good statistical practice in pharmacology: problem 2. Br. J. Pharmacol..

[69] Krämer A, Green J, Pollard J, Tugendreich S (2014). Causal analysis approaches in Ingenuity Pathway Analysis. Bioinformatics.

