# Publisher Correction: Computational repositioning and preclinical validation of mifepristone for human vestibular schwannoma

**DOI:** 10.1038/s41598-018-36016-9

**Published:** 2018-11-23

**Authors:** Jessica E. Sagers, Adam S. Brown, Sasa Vasilijic, Rebecca M. Lewis, Mehmet I. Sahin, Lukas D. Landegger, Roy H. Perlis, Isaac S. Kohane, D. Bradley Welling, Chirag J. Patel, Konstantina M. Stankovic

**Affiliations:** 1 Eaton-Peabody Laboratories, Department of Otolaryngology, Massachusetts Eye and Ear, Boston, MA 02114 USA; 2 Program in Speech and Hearing Bioscience and Technology, Harvard Medical School, Boston, MA 02115 USA; 3 Department of Biomedical Informatics, Harvard Medical School, Boston, MA 02115 USA; 4 Harvard Program in Therapeutic Science, Harvard Medical School, Boston, MA 02115 USA; 5 Department of Otolaryngology, Harvard Medical School, Boston, MA 02114 USA; 6 Department of Otolaryngology, Vienna General Hospital, Medical University of Vienna, Vienna, 1090 Austria; 7 Center for Experimental Drugs and Diagnostics, Department of Psychiatry and Center for Human Genetic Research, Massachusetts General Hospital, Harvard Medical School, Boston, MA 02114 USA

Correction to: *Scientific Reports* 10.1038/s41598-018-23609-7, published online 03 April 2018

In the original version of this Article the author Rebecca M. Lewis was incorrectly indexed. This error has now been corrected in the PDF and HTML versions of the article.

