# Supplementary material for: Computational repositioning and preclinical validation of mifepristone for human vestibular schwannoma
Source: Sci Rep. 2018 Apr 3;8:5437. doi: 10.1038/s41598-018-23609-7 (PMC5882888; doi:10.1038/s41598-018-23609-7)
Supplement: Supplementary file 1 — Supplementary Information [file 41598_2018_23609_MOESM1_ESM.pdf]

Supplementary Information

Computational Repositioning and Preclinical Validation of Mifepristone for Human Vestibular Schwannoma

Jessica E. Sagers, Adam S. Brown, Sasa Vasilijic, Rebecca Lewis, Mehmet I. Sahin, Lukas D. Landegger, Roy H. Perlis, Isaac S. Kohane, D. Bradley Welling, Chirag J. Patel, Konstantina M. Stankovic

Data File and Video Captions

Supplementary Data File S1. CSV file containing meta-analysis of 80 VSs and 16 control nerves. Gene symbols, p values describing differential gene expression in each dataset, and Bonferroni-corrected meta-analytic p values are provided.

Supplementary Data File S2. CSV file containing full ksRepo output for the complete meta-analysis.

Supplementary Data File S3. CSV file containing combined analysis of 13 NF2-associated tumors. Gene symbols, p values describing differential gene expression in each dataset, and Bonferroni-corrected meta-analytic p values are provided.

Supplementary Data File S4. CSV file containing full ksRepo output for the NF2-associated tumor meta-analysis.

Supplementary Video S1. Time-lapse phase contrast image set of HEI-193 cells treated with DMSO vehicle (0.1% in culture medium). Live cell images acquired every 2 h for 60 h in culture using an IncuCyte S3 instrument (10X magnification). Vehicle-treated cells are seen to proliferate normally.

Supplementary Video S2. Time-lapse phase contrast image set of HEI-193 cells treated with 35 μM mifepristone in culture medium. Live cell images acquired every 2 h for 60 h in culture using an IncuCyte S3 instrument (10X magnification). Mifepristone-treated cells are seen to stretch into an elongated shape and do not proliferate normally.

Supplementary Figure S1

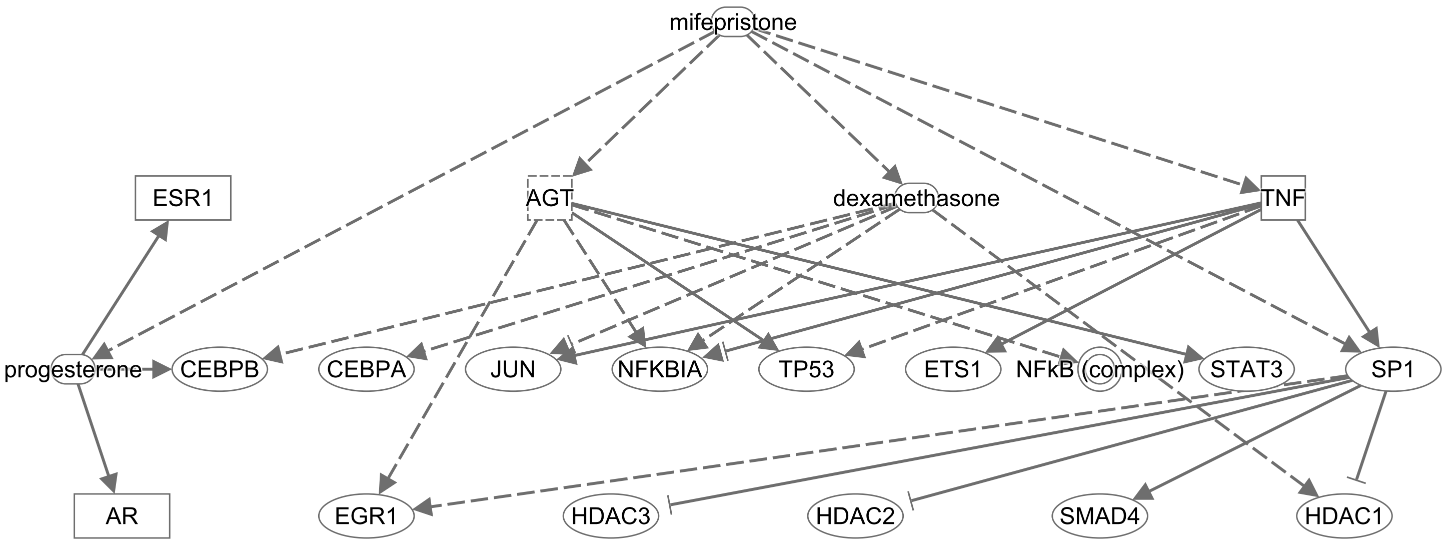

Supplementary Figure S1. Ingenuity Pathway Analysis (QIAGEN Inc., https://www.qiagenbioinformatics.com/products/ingenuitypathway-analysis)[69] highlights mifepristone as a significant upstream regulator of predicted regulatory networks generated after the analysis of all genes in the 80-tumor meta-analysis (**Supplementary Data File S1**) that were identified as significantly differentially regulated after Bonferroni correction for multiple hypothesis testing ($p=4.26*10^{-5}$). Dotted lines, theorized relationships; solid lines, known relationships.

Supplementary Figure S2

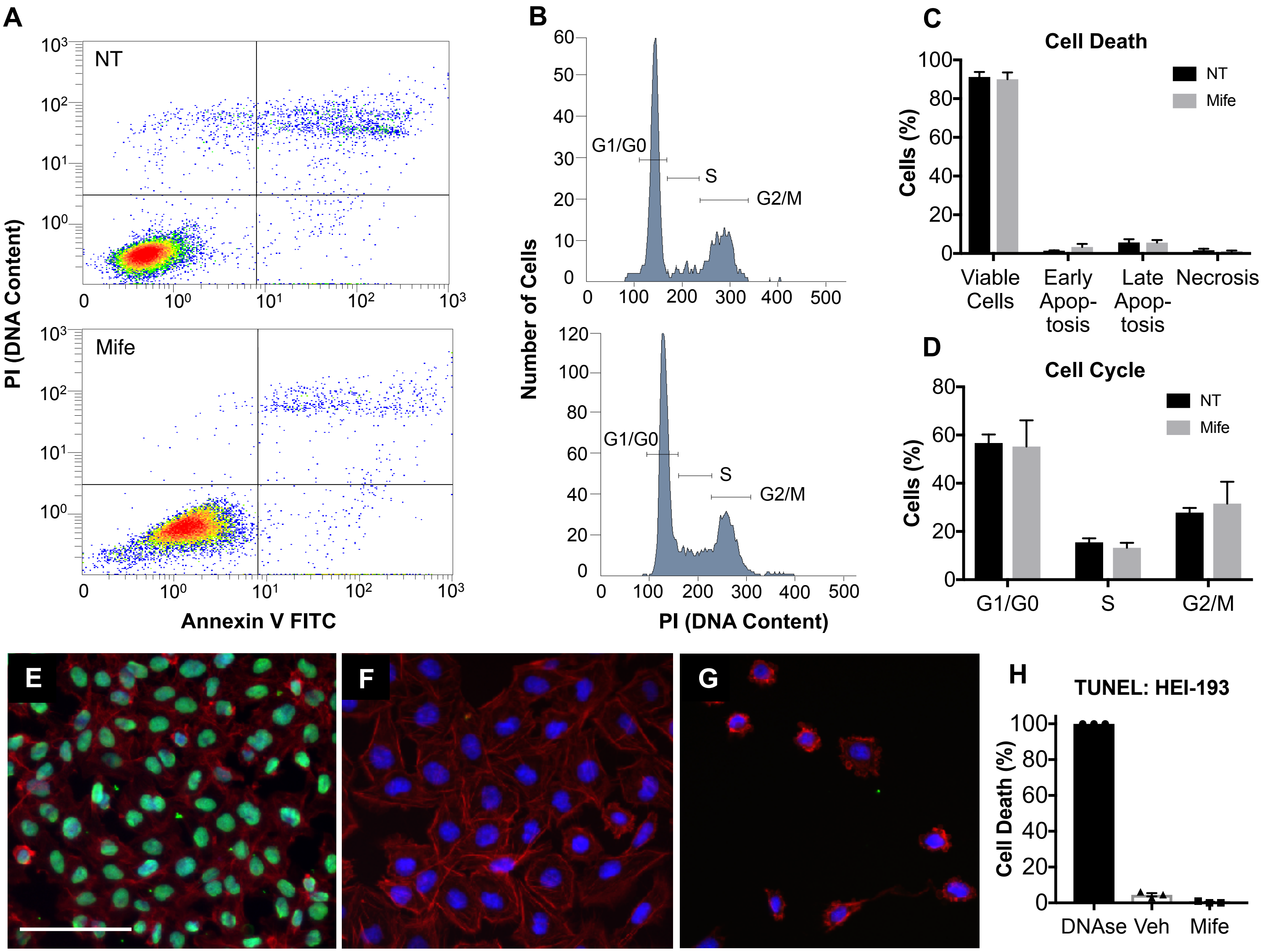

Supplementary Figure S2. Flow cytometry for annexin V/propidium iodide staining and terminal deoxynucleotidyl transferase dUTP nick-end labeling (TUNEL) assay reveal no significant differences in the apoptotic cell fraction or phase of cell cycle in mifepristone-treated cells versus vehicle-treated controls. **A**, Annexin V and propidium iodide labeling of HEI-193 cells treated with 35 μM mifepristone for 72 h reveals a slight but not significant increase in

early apoptotic cells as compared to vehicle-treated controls (quantified in **C**). **B**, cell cycle analysis exhibits no significant differences in phase of cell cycle after 72 h mifepristone treatment (quantified in **D**). **E-H**, TUNEL assay reveals no statistically significant difference between the number of TUNEL-positive cells in the mifepristone-treated and vehicle-treated conditions (representative results; experiment repeated three times in duplicate, quantifying three fields of view per treatment condition): **E**, DNAse-treated positive control; **F**, TUNEL stain on cells treated with 0.1% DMSO vehicle; **G**, TUNEL stain on cells treated with 35 μM mifepristone for 72 h.; green, TUNEL; blue, Hoechst stain; red, rhodamine phalloidin. **H**, quantification of TUNEL assay (n=3).

Supplementary Figure S3

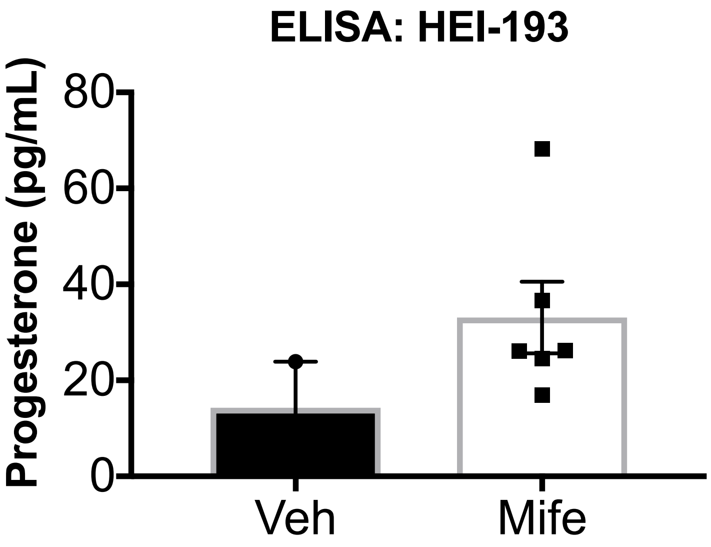

Supplementary Figure S3. Enzyme-linked immunosorbent assay (ELISA) on conditioned cell culture medium collected from HEI-193 cells treated with 35 µM mifepristone and 0.1% DMSO vehicle (n=6 from mifepristone-treated cells, 2 from vehicle-treated cells). Mifepristone-treated cells showed an increase in progesterone in culture medium, suggesting that the drug is effectively competing with progesterone for receptor binding (two-tailed unpaired T test, p=0.08).

Supplementary Figure S4

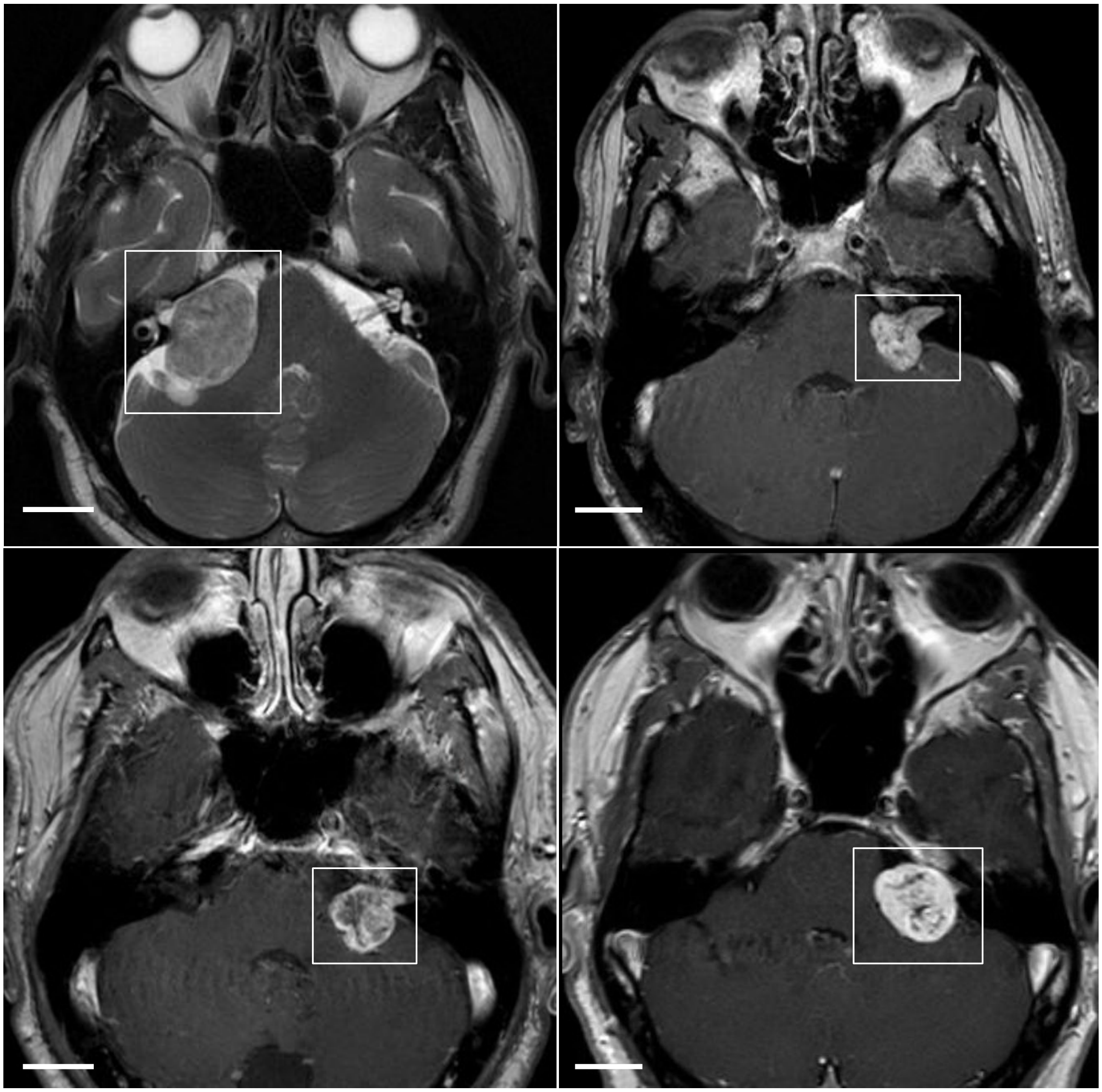

Supplementary Figure S4. Four additional MRI scans of VS patients whose primary tumor cells were treated with mifepristone after surgical resection. White rectangles, VSs; scale bars, 20 mm.

Supplementary Methods

Computational Repositioning and Preclinical Validation of Mifepristone for Human Vestibular Schwannoma

Jessica E. Sagers, Adam S. Brown, Sasa Vasilijic, Rebecca Lewis, Mehmet I. Sahin, Lukas D. Landegger, Roy H. Perlis, Isaac S. Kohane, D. Bradley Welling, Chirag J. Patel, Konstantina M. Stankovic

Experimental Design

The objectives of this study were to collect the maximum possible number of complete VS transcriptomes as measured by microarray and subject these to meta-analysis (n = 80 VSs); to screen resulting differential gene expression data against publicly available gene-drug interactions; and to test the most promising candidates on immortalized human schwannoma cells, immortalized human arachnoid cells, and primary VS cells harvested from patients (10 VS patients and 8 patients undergoing unrelated surgery that required removal of the healthy great auricular nerve (GAN); detailed consent, IRB, and OR collection methods described below). For the generation of primary human VS and Schwann cells, inclusion criteria were prospectively established: all human subjects who visited our hospital for surgery during the experimental period were offered the opportunity to give informed consent and participate in the study. No discrimination was made on the basis of tumor size, gender, ethnicity, age, or race. No obvious outliers were observed and all data are included in the paper. Replication and internal controls for each experiment are described in detail. Experiments performed on immortalized human schwannoma and immortalized arachnoid cell lines were conducted on multiple thawed aliquots of cells at low passage numbers (<20) to ensure reproducibility. Mycoplasma testing of cells was conducted and confirmed to be negative. Metabolic activity, proliferation, confluence, and cytotoxicity assays performed on primary cells and cell lines were performed on 3-5 wells of cells per treatment per tumor to ensure maximal statistical power. Quantification of immunocytochemical results was performed blind, as described in detail. All methods for genomic and transcriptomic analysis are presented in detail and all datasets are made publicly available for external investigators to verify processing, analysis, and interpretation.

Detailed Methods: VS and GAN Cell Culture

In accordance with published protocols[37,38], surgical VS and GAN specimens were transported to the laboratory on ice. Specimens were rinsed with Hank's Balanced Salt Solution (HBSS, ThermoFisher Scientific), dissected to remove burned tissue and blood vessels, and separated for RNA preservation (RNALater, ThermoFisher Scientific) or primary cell culture. After enzymatic dissolution (collagenase type I, 160 U/mL; hyaluronidase type I-S, 250 U/mL) and trituration with an 18-gauge needle, primary cell culture suspensions were plated on 12 mm coverslips pre-coated with poly-D-lysine and laminin (Neuvitro) and grown in Dulbecco's Modified Eagle's Medium (DMEM) and F12-containing medium (ThermoFisher Scientific) consisting of 44.5% DMEM, 44.5% F12 nutrient mixture, 10% fetal bovine serum (ThermoFisher Scientific), and 1% of a mixture of penicillin and streptomycin (ThermoFisher Scientific). VS and GAN cultures were incubated at 37 degrees Celsius with 5% carbon dioxide, and culture medium was changed every three days.

Detailed Methods: Library Preparation and Targeted Capture

Briefly, 50 ng of genomic DNA from each sample diluted with nuclease-free water to a final concentration of 1.8 ng/µL were digested in eight different reactions, each containing two restriction enzymes. Successful digestion of ECD gDNA was indicated by the appearance of three predominant bands at 124, 255, and 450 bp, corresponding to the 800-bp PCR product-derived restriction fragments. A library of HaloPlex probes designed using the HaloPlex SureDesign program (www.genomics.agilent.com) was hybridized to the library of genomic DNA restriction fragments. Enrichment was validated by gel electrophoresis. Following purification, the DNA concentration of each library was quantified using the high-sensitivity D1000 DNA Tapescreen analysis assay on the Tapestation 2200 instrument (Agilent Technologies), and samples were subsequently sequenced.

Detailed Methods: Bioinformatic Processing and Variant Prioritization of NF2 *Gene Sequencing Data*

The quality criteria for MiSeq includes a number of generated clusters between 600 and 1200 K/mm^2, >90% passed filter clusters, and approximately 5% sequenced ECD. To be included in the analysis, bases had at least a quality score of 40, and depth of coverage was at least 100 for all samples. Raw data were demultiplexed and converted to fastq using Illumina bcl2fastq conversion software (v 2.16.0.10) as directed by Agilent and Illumina. Prior to alignment, Agilent AGeNT (v3.5.1.46) was used to trim low-quality bases from the ends, remove adaptor sequences, and remove duplicated reads based on Molecular Barcode information following Agilent directions. Alignment was done by BWA (Burrows-Wheeler Aligner v0.7.13) "mem" algorithm using UCSC hg19 Human Reference Genome, variants and indels were *called* using GATK (Genome Analysis Toolkit v3.5) following best practices, choosing *HaplotypeCaller* to generate a joint called Variant Call Format (VCF) file for all samples.

Genomic variant annotation was performed using ANNOVAR (ANNOtate VARiation v2016-02-01). A filter was applied to eliminate common variants as reported in the 1000 Genomes database. Data were visualized using the Integrative Genomics Viewer (IGV; Broad Institute, Cambridge, MA), and used to identify rare variants. To confirm accuracy of the sequencing read for rare variants, individual sample BAM files were visualized in the IGV software and analyzed for potential errors in sequencing. Using the 2017 release of the gnomAD browser (Broad Institute, Cambridge, MA), which contains exome sequence data from 123,136 individuals and whole genome sequencing from 15,496 individuals, remaining filtered variants were probed for previous reports in the literature. The Single Nucleotide Polymorphism database (dbSNP) was also referenced to determine whether rare variants identified by the gnomAD and

1000 Genomes databases were either novel or previously reported using this public-domain archive.
